# The host microbiota is associated with the occurrence and development of esophageal squamous cell carcinoma

**DOI:** 10.1093/procel/pwac024

**Published:** 2022-08-23

**Authors:** Jing Zuo, Yanjun Liu, Wenjing Lv, Yudong Wang, Zhisong Fan, Long Wang, Li Feng, Xue Zhang, Jing Han, Zhiyu Ni

**Affiliations:** Department of Oncology, The Fourth Hospital of Hebei Medical University, Shijiazhuang 050011, China; Department of Oncology, The Fourth Hospital of Hebei Medical University, Shijiazhuang 050011, China; Department of Oncology, The Fourth Hospital of Hebei Medical University, Shijiazhuang 050011, China; Department of Oncology, The Fourth Hospital of Hebei Medical University, Shijiazhuang 050011, China; Department of Oncology, The Fourth Hospital of Hebei Medical University, Shijiazhuang 050011, China; Department of Oncology, The Fourth Hospital of Hebei Medical University, Shijiazhuang 050011, China; Department of Oncology, The Fourth Hospital of Hebei Medical University, Shijiazhuang 050011, China; Department of Oncology, The Fourth Hospital of Hebei Medical University, Shijiazhuang 050011, China; Department of Oncology, The Fourth Hospital of Hebei Medical University, Shijiazhuang 050011, China; The Affiliated Hospital of Hebei University, Baoding 071000, China; School of Basic Medical Science, Hebei University, Baoding 071000, China

**Dear Editor,**

Microecology is an emerging research area, particularly that of the digestive system (Marchesi et al., 2016; Wang et al., 2018). The intestinal microbiota contains a rich and complex microbial ecosystem that is in dynamic balance, the disruption of which can result in disease (Yatsunenko et al., 2012; Li et al., 2017; He et al., 2018). For example, studies have found that the esophagus has a variety of colonizing bacteria that may be related to esophageal cancer.

Esophageal cancer is the seventh most common cancer in the world (Sung et al., 2021). Esophageal squamous cell carcinoma (ESCC) has a 5-year survival rate less than 20% (May et al., 2018). Although the mechanisms underlying ESCC are not completely known, ESCC is associated with certain bacteria colonizing the esophagus (Shao et al., 2019). Metagenomics research and the development of 16S rRNA gene sequencing technology have allowed the study of the composition of esophageal microbial communities. Furthermore, because saliva, oral mucosa, feces, blood, and urine contain microbes in healthy people (Eckburg et al., 2005; Chen et al., 2015; Poore et al., 2020), we hypothesized that abnormal microbial communities in these tissues may be associated with the occurrence and development of ESCC, and that the microbial communities of ESCC patients will change after treatment. In this study, we compared the microbial communities in saliva, oral mucosa, feces, blood, and urine between ESCC patients and healthy subjects. We also compared the microbial communities in these tissues in ESCC patients who received either chemotherapy or immunotherapy.

We analyzed the microbiota composition of blood, oral mucosa, saliva, urine, and fecal samples from ESCC patients and healthy subjects at the phylum and genus levels (Fig. S1). We found that the microbial composition of blood and urine samples was similar in ESCC patients, as both tissues included high counts of *Cupriavidus* and *Vogesella*. However, these microbial genera were either absent or present at low levels in healthy subjects, suggesting that the microbiota is different between ESCC patients and healthy people, and that the microbiota is associated with ESCC. Taken together, these results suggest that microbes may be a biomarker for ESCC.

To compare the richness and diversity of microbial compositions between ESCC patients and healthy subjects, we compared the alpha diversity values of the samples. To characterize richness, we used Chao1 and observed species indices. We also used Shannon and Simpson indices to characterize diversity and Faith’s PD index to characterize diversity based on evolution. Uniformity was represented by Pielou’s evenness index and the coverage was represented by Good’s coverage index. In blood samples, the Shannon index was higher in healthy subjects than in ESCC patients (*P* = 0.00017, Kruskal–Wallis and Dunn’s test; Figs. 1A and S2A). In oral mucosal samples, the Faith index was higher in healthy subjects than in ESCC patients (*P* = 0.001, Kruskal–Wallis and Dunn’s test; Figs. 1B and S2B). In saliva samples, the Chao1 index was higher in healthy subjects than in ESCC patients (*P* = 0.047, Kruskal–Wallis and Dunn’s test), as was the Faith index (*P* = 0.025, Kruskal–Wallis and Dunn’s test) and Good’s coverage index (*P* = 0.00036, Kruskal–Wallis and Dunn’s test; Figs. 1C and S2C). In urine samples, the Shannon index was higher in healthy subjects than in ESCC patients (*P* = 0.00047, Kruskal–Wallis and Dunn’s test; Figs. 1D and S2D). Finally, in fecal samples, the Chao1 index was higher in healthy subjects than in ESCC patients (*P* = 0.012, Kruskal–Wallis and Dunn’s test), as was Good’s coverage index (*P* = 0.00072, Kruskal–Wallis and Dunn’s test; Figs. 1E and S2E). Taken together, these results suggest that the diversity, richness, and coverage of the microbial communities are higher in healthy subjects than in ESCC patients. Intriguingly, the diversity of intestinal microbes is also reduced in patients with ulcerative colitis, Crohn’s disease, and colon cancer (Chassaing et al., 2011; Marchesi et al., 2011). This suggests that the occurrence and development of gastrointestinal diseases may be closely related to the decline of microbial diversity.

**Figure 1.**
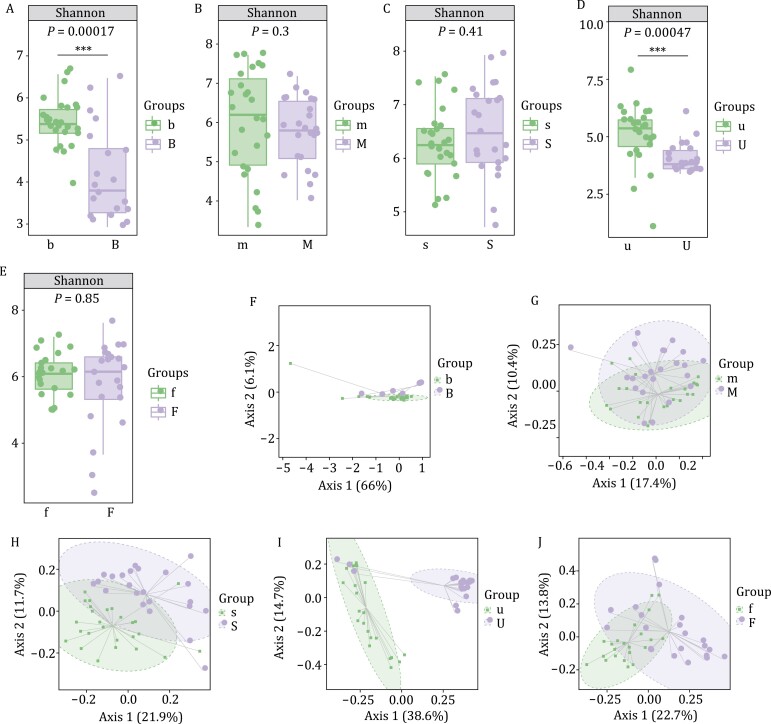
**The microbial compositions of samples from ESCC patients and healthy subjects differ substantially.** (A) The Shannon index values were higher in blood samples from healthy subjects than in those from ESCC patients (*P* < 0.05, Kruskal–Wallis and Dunn’s test). (B) The Shannon index values were higher in oral mucosal samples from healthy subjects than in those from ESCC patients (*P* > 0.05, Kruskal–Wallis and Dunn’s test). (C) The Shannon index values were lower in saliva samples from healthy subjects than in those from ESCC patients (*P* > 0.05, Kruskal–Wallis and Dunn’s test). (D) The Shannon index values were higher in urine samples from healthy subjects than in those from ESCC patients (*P* < 0.05, Kruskal–Wallis and Dunn’s test). (E) The Shannon index values were lower in fecal samples from healthy subjects compared to those from ESCC patients (*P* > 0.05, Kruskal–Wallis and Dunn’s test). β diversity values were different (*P* < 0.05, Adonis test) between ESCC patients and healthy subjects in (F) blood, (G) oral mucosal, (H) saliva, (I) urine, and (J) fecal samples. The number of biological replicates performed was as follows: in ESCC patients, B (*n* = 20), M (*n* = 24), S (*n* = 23), U (*n* = 21), F (*n* = 22); in healthy subjects, b (*n* = 27), m (*n* = 26), s (*n* = 26), u (*n* = 25), f (*n* = 22).

We also compared the compositions of the microbial communities (i.e., β diversity) in samples between ESCC patients and healthy subjects. We identified significantly different clusters of microbial-weighted UniFrac distances between healthy subjects and ESCC patients in blood, oral mucosa, saliva, urine, and fecal samples (*P* < 0.05, Adonis test, Fig. 1F–J). All these indicate that the bacterial flora of ESCC patients has changed to some extent after different treatments.

To explore the functions of the microbiota in ESCC patients and healthy subjects, we compared the microbial functions in ESCC patients and healthy subjects using PICRUSt2. The relative abundance of microbial metabolic pathways in ESCC patients was like that of healthy patients (37,503 vs. 37,418; Fig. S3A and S3B). In both ESCC patients and healthy subjects, metabolic pathways accounted for the greatest number of microbial functions.

A previous study detected 10 KEGG pathways that were significantly overrepresented in *F. nucleatum*-positive EC tissues (Yamamura et al., 2016). We predicted that these metabolic pathways would be different in samples from ESCC patients and healthy patients. A comparison of the metabolic pathways in blood samples revealed that 84 microbial metabolic pathways were overrepresented in ESCC patients, whereas 20 were underrepresented (*P* < 0.05; Fig. S3C). In oral mucosal samples, only the betalain biosynthesis metabolic pathway was overrepresented (*P* < 0.001; Fig. S3D). In saliva samples, betalain biosynthesis and limonene and pinene degradation metabolic pathways were overrepresented (*P* < 0.1; Fig. S3E), while in urine samples, indole–alkaloid biosynthesis was overrepresented (*P* < 0.01; Fig. S3F). However, there were no differences in the KEGG pathways in fecal samples. While there were obvious differences in the metabolic pathways represented in blood samples, oral samples, and saliva samples in ESCC patients compared to healthy patients, there were no differences in fecal samples. Because blood samples are easy to collect, identifying microbial metabolic pathways associated with ESCC in blood could lead to the discovery of biomarkers for ESCC.

We analyzed the microbiota composition of samples from ESCC patients after either chemotherapy or immunotherapy at the phylum and genus levels (Fig. S4). At the genus level, *Aquabacterium* and *Lactobacillus* were widely present in samples from ESCC patients after immunotherapy, while samples from ESCC patients who received chemotherapy mainly consisted of *Cupriavidus* and *Vogesella* (Fig. S4). However, how these genera affect the prognosis of patients still needs to be researched.

We compared the alpha diversity values between tissue samples from ESCC patients after chemotherapy or immunotherapy. In blood samples, the Faith’s PD index of patients after immunotherapy was higher than that after chemotherapy (*P* = 0.0067, Kruskal–Wallis and Dunn’s test; Figs. 2A and S5A). In oral mucosal samples, the Good’s coverage index of patients was higher after immunotherapy than after chemotherapy (*P* = 7.1 × 10^−5^, Kruskal–Wallis and Dunn’s test). The Chao1 index (*P* = 0.0017, Kruskal–Wallis and Dunn’s test) and the observed species index (*P* = 0.012, Kruskal–Wallis and Dunn’s test) was less after immunotherapy than after chemotherapy (Figs. 2B and S5B). In urine samples, the Chao1 index of patients who received immunotherapy was significantly higher than after chemotherapy (*P* = 8.1 × 10^−5^, Kruskal–Wallis and Dunn’s test), as was the Shannon index (*P* = 0.0082, Kruskal–Wallis and Dunn’s test), the Pielou index (*P* = 0.0039, Kruskal–Wallis and Dunn’s test), the observed species index (*P* = 8.1 × 10^−5^, Kruskal–Wallis and Dunn’s test), and the Faith index (*P* = 0.00056, Kruskal–Wallis and Dunn’s test) (Figs. 2D and S2D). In saliva samples and fecal samples, there was no statistically significant difference between ESCC patients receiving immunotherapy or chemotherapy (Figs. 2C, 2E, S2C, and S2E). The microbial diversity of patients after immunotherapy was higher than that after chemotherapy.

**Figure 2.**
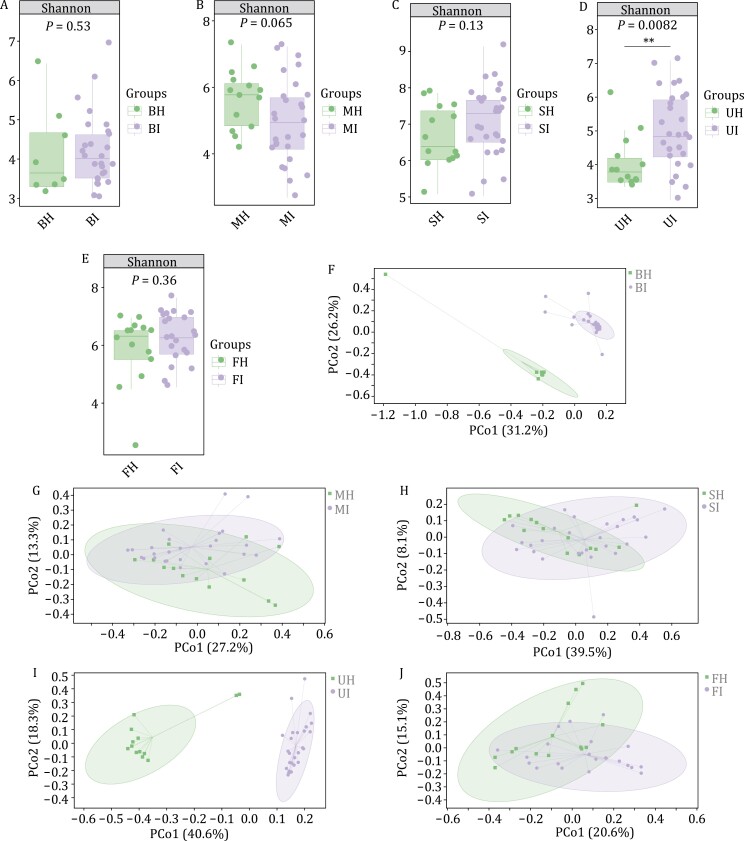
**The microbial compositions of samples from ESCC patients after chemotherapy and ESCC patients after immunotherapy differ substantially.** (A) The Shannon index values were higher in blood samples from patients with ESCC after chemotherapy than in those from patients with ESCC after immunotherapy (*P* > 0.05, Kruskal–Wallis and Dunn’s test). (B) The Shannon index values were higher in oral mucosal samples from patients with ESCC after chemotherapy than in those from patients with ESCC after immunotherapy (*P* > 0.05, Kruskal–Wallis and Dunn’s test). (C) The Shannon index values were lower in saliva samples from patients with ESCC after chemotherapy than in those from patients with ESCC after immunotherapy (*P* > 0.05, Kruskal–Wallis and Dunn’s test). (D) The Shannon index values were lower in urine samples from patients with ESCC after chemotherapy than in those from patients with ESCC after immunotherapy (*P* < 0.05, Kruskal–Wallis and Dunn’s test). (E) The Shannon index values were lower in fecal samples from patients with ESCC after chemotherapy than in those from patients with ESCC after immunotherapy (*P* > 0.05, Kruskal–Wallis and Dunn’s test). β diversity values were different (*P* < 0.05, Adonis test) between patients with ESCC after chemotherapy and patients with ESCC after immunotherapy in (F) blood, (G) oral mucosal, (H) saliva, (I) urine, and (J) fecal samples. The number of biological replicates is as follows: in patients with ESCC after chemotherapy, BH (*n* = 8), MH (*n* = 14), SH (*n* = 14), UH (*n* = 13), FH (*n* = 14); in patients with ESCC after immunotherapy, BI (*n* = 27), MI (*n* = 27), SI (*n* = 27), UI (*n* = 27), FI (*n* = 22).

The microbial-weighted UniFrac distances were statistically different between patients after chemotherapy or immunotherapy in blood, oral mucosal, saliva, urine, and fecal samples (*P* < 0.05, Adonis test, Fig. 2F–J). A comparison of the α and β diversities revealed that the microbial diversity of patients after immunotherapy was higher than after chemotherapy. Previous studies have shown that chemotherapy changes the composition of the microbiota in the small intestine and induces metastasis of selected species of Gram-positive bacteria to secondary lymphoid organs, which stimulate the production of specific immune responses (Viaud et al., 2013).

We also predicted microbial function in ESCC patients after chemotherapy or immunotherapy using PICRUSt2. The relative abundance of microbial metabolic pathways in patients after immunotherapy was similar to that in patients after chemotherapy (29,708.5 vs. 29,729.7; Fig. S6A and S6B).

To detect whether there were differences in microbial metabolic pathways in ESCC patients after chemotherapy or immunotherapy, we compared KEGG pathways. Comparison of metabolic pathways in blood samples revealed that 8 microbial metabolic pathways were overrepresented in patients after chemotherapy, whereas 13 were underrepresented (*P* < 0.05; Fig. S6C). In urine samples, 17 microbial metabolic pathways were overrepresented in patients after chemotherapy, whereas 10 were underrepresented (*P* < 0.05; Fig. S6F). In oral mucosal samples, the polyketide sugar unit biosynthesis and betalain biosynthesis metabolic pathways were underrepresented in patients after immunotherapy (*P* < 0.05; Fig. S6D). Comparison of metabolic pathways in saliva samples revealed that photosynthesis and lysosome metabolic pathways were overrepresented in patients after immunotherapy compared with patients after chemotherapy (*P* < 0.05; Fig. S6E). In fecal samples, tetracycline biosynthesis, lysosome, and shigellosis metabolic pathways were overrepresented in patients after immunotherapy (*P* < 0.05; Fig. S6G). Interestingly, we found that metabolic pathways diverged in blood and urine more often than in other tissue types.

In summary, our results indicated that ESCC patients had different microbial content in blood, oral mucosa, saliva, urine, and feces compared to healthy subjects. Furthermore, the species and quantity of the microbiota in blood, oral mucosa, saliva, urine, and feces of ESCC patients after chemotherapy were also different from ESCC patients who received immunotherapy. While the species and quantity of microbiota differed depending upon the tissue type and mechanism, and clinical application is still lacking, an advantage of all the sample types examined is that they are easily obtained and avoid damage to the esophagus. The abnormal microbial communities associated with ESCC could be used as clinical indicators to diagnose ESCC. Furthermore, if we study these microorganisms to discover how they affect ESCC treatment, we may be able to improve patient prognosis by applying antibiotics targeting the corresponding flora.

## Supplementary Material

pwac024_suppl_Supplementary_Material_S1

pwac024_suppl_Supplementary_Material_S2

## Footnotes

This work was supported by the Hebei Natural Science Foundation Outstanding Youth Fund (No. H2017206332), grants from CAMS Innovation Fund for Medical Sciences (No. 2019-I2M-5-055). We thank Shanghai Personal Biotechnology Co., Ltd. (Shanghai, China) for technical support. Jing Zuo, Yanjun Liu, Wenjing Lv, Yudong Wang, Zhisong Fan, Long Wang, Li Feng, Xue Zhang, Jing Han and Zhiyu Ni declare that they have no conflict of interest. J.Z. and Z.Y.N. conceived the study and supervised the project. J.Z. and Y.J.L. wrote the manuscript. W.J.L., L.W., and J.H. collected the samples. L.F. and X.Z. analyzed the data. W.J.L., Y-D.W. and Z.S.F. edited and proofread the manuscript. All authors read and approved the final manuscript.
