# Supplementary material for: The host microbiota is associated with the occurrence and development of esophageal squamous cell carcinoma
Source: Protein Cell. 2022 Aug 23;14(4):294–8. doi: 10.1093/procel/pwac024 (PMC10120988; doi:10.1093/procel/pwac024)
Supplement: pwac024_suppl_Supplementary_Material_S1 [file pwac024_suppl_supplementary_material_s1.pdf]

Supplemental materials

Materials and Methods

Procedure for Sample Collection

Twenty-six ESCC patients, fourteen patients with ESCC after chemotherapy, eighteen patients with ESCC after immunotherapy and twenty-eight healthy volunteers were enrolled in this study. Blood, oral mucosal, saliva, urine, and fecal samples were collected once from the subjects. We used blood test tubes to collect the blood samples and urinary tubes to collect the urine samples and transferred the samples to sterile EP tubes. Oral mucosal, saliva, and fecal samples were collected using sterile sampling tubes. Oral mucosal and saliva samples were collected from subjects in the morning after they had brushed their teeth and gargled. We collected the middle part of the morning urine and the inside part of the middle feces. Samples were stored at −80 °C until analysis.

DNA Extraction

Total genomic DNA samples were extracted using the OMEGA Soil DNA Kit (M5635-02) (Omega Bio-Tek, Norcross, GA, USA), following the manufacturer's instructions, and stored at -20 ℃ prior to further analysis. The quantity and quality of extracted DNAs were measured using a NanoDrop NC2000 spectrophotometer (Thermo Fisher Scientific, Waltham, MA, USA) and agarose gel electrophoresis, respectively.

16S rRNA Gene Amplicon Sequencing

PCR amplification of the bacterial 16S rRNA genes V3–V4 region was performed using the forward primer 338F (5'-ACTCCTACGGGAGGCAGCA-3') and the reverse primer 806R (5'-GGACTACHVGGGTWTCTAAT-3'). Sample-specific 7-bp barcodes were incorporated into the primers for multiplex sequencing. The PCR components contained 5 μl of buffer (5×), 0.25 μl of Fast pfu DNA Polymerase (5U/μl), 2 μl (2.5 mM) of dNTPs, 1 μl (10 uM) of each Forward and Reverse primer, 1 μl of DNA Template, and 14.75 μl of ddH2O. Thermal cycling consisted of initial denaturation at 98 ℃ for 5 min, followed by 25 cycles consisting of denaturation at 98 ℃ for 30 s, annealing at 52 ℃ for 30 s, and extension at 72 ℃ for 45 s, with a final extension of 5 min at 72 ℃. PCR amplicons were purified with Vazyme VAHTSTM DNA Clean Beads (Vazyme, Nanjing, China) and quantified using the Quant-iT PicoGreen dsDNA Assay Kit (Invitrogen, Carlsbad, CA, USA). After the individual quantification step, amplicons were pooled in equal amounts, and pair-end 2×250 bp sequencing was performed using the Illlumina NovaSeq platform with NovaSeq 6000 SP Reagent Kit (500 cycles) at Shanghai Personal Biotechnology Co., Ltd (Shanghai, China).

Sequence Analysis

Microbiome bioinformatics were performed with QIIME2 2019.4 (Bolyen et al. 2018) with slight modification according to the official tutorials

(https://docs.qiime2.org/2019.4/tutorials/). Briefly, raw sequence data were demultiplexed using the demux plugin following by primers cutting with cutadapt plugin (Martin, M., 2011). Sequences were then quality filtered, denoised, merged and chimera removed using the DADA2 plugin (Callahan et al. 2016). Non-singleton amplicon sequence variants (ASVs) were aligned with mafft (Katoh et al. 2002) and used to construct a phylogeny with fasttree2 (Price et al. 2010). Alpha-diversity metrics (Chao1 (Chao, 1984), Observed species, Shannon (Shannon, 1948a, b), Simpson (Simpson, 1949), Faith’s PD (Faith, 1992), Pielou’s evenness (Pielou, 1966) and Good’s coverage (Good, 1953)), beta diversity metrics weighted UniFrac (Lozupone et al. 2007) was estimated using the diversity plugin with samples were rarefied to 22377 sequences per sample. Taxonomy was assigned to ASVs using the classify-sklearn naïve Bayes taxonomy classifier in feature-classifier plugin (Bokulich et al. 2018a) against the Greengenes Release 13.8 Database (DeSantis et al,2006).

Bioinformatics and Statistical Analysis

Sequence data analyses were mainly performed using QIIME2 and R packages (v3.2.0). ASV-level alpha diversity indices, such as Chao1 richness estimator, Observed species, Shannon diversity index, Simpson index, Faith’s PD, Pielou’s evenness and Good’s coverage were calculated using the ASV table in QIIME2, and visualized as box plots. Beta diversity analysis was performed to investigate the structural variation of microbial communities across samples using UniFrac distance

metrics (Lozupone and Knight 2005, Lozupone, Hamady et al. 2007) and visualized via principal coordinate analysis (PCoA).

Functional Prediction of the Microbiota

Microbial functions were predicted by PICRUSt2 (Phylogenetic investigation of communities by reconstruction of unobserved states) (Gavin M. Douglas, et al., preprint) upon KEGG (https://www.kegg.jp/) databases.

Accession numbers

Bacterial 16S rRNA gene sequencing data has been deposited in NCBI (BioProject ID: PRJNA626546).

Supplemental information

Figure S1. The microbial compositions of samples from ESCC patients and healthy subjects. (A) The microbial composition of blood, oral mucosal, saliva, urine, and fecal samples at the genus level in ESCC patients. (B) The microbial composition of blood (b), oral mucosal, saliva, urine, and fecal samples at the genus level in healthy subjects. (C)The composition of microbiota in the blood, oral mucosa, saliva, urine, and feces of ESCC patients at the phylum level; the top 20 microbial types at the phylum level in ESCC patients are shown. (B) The composition of the microbiota in the blood, oral mucosa, saliva, urine, and feces of healthy subjects at the phylum level; the top 20 microbial types at the phylum level in healthy subjects are shown. The number of biological replicates performed was as follows: in ESCC patients, BT (*n* =20), MT (*n*=24), ST (*n*=23), UT (*n*=21), FT (*n*=22); in healthy subjects, b (*n* =27), m (*n*=26), s (*n*=26), u (*n*=25), f (*n*=22).

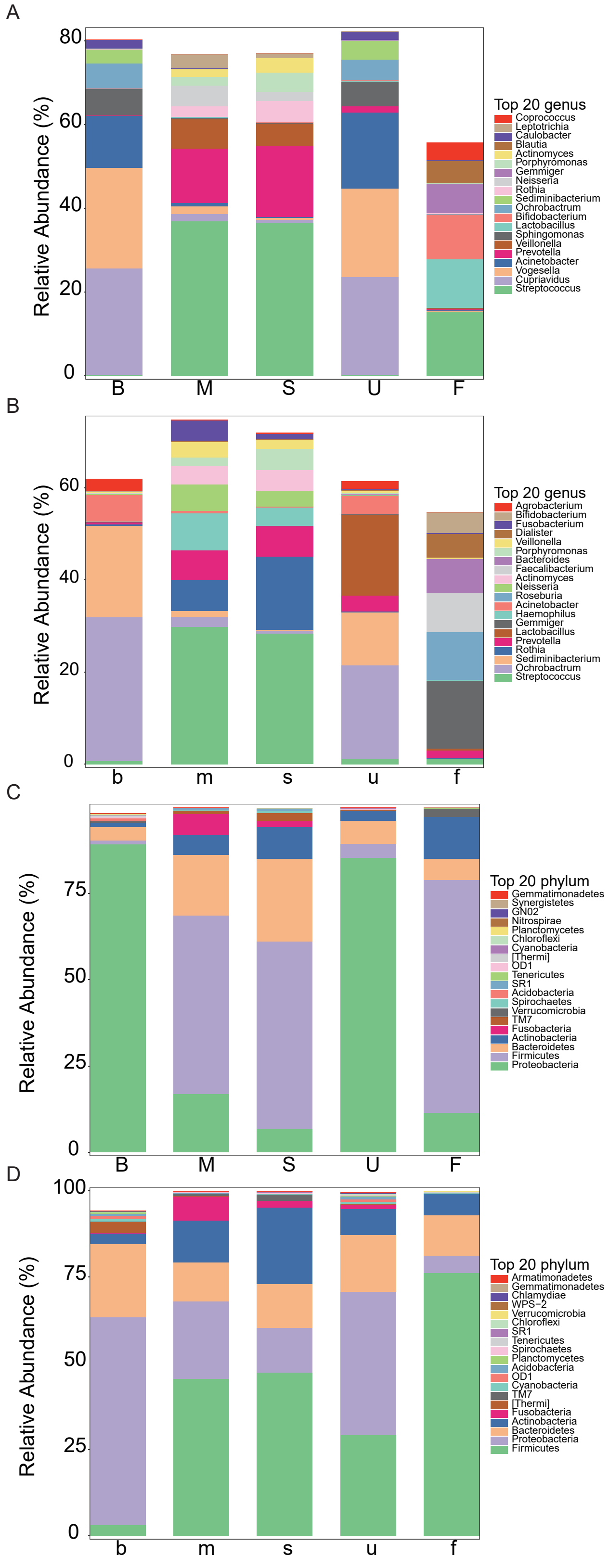

A
Relative Abundance (%)
0
20
40
60
80
B
M
S
U
F
Top 20 genus
Coprococcus
Leptotrichia
Caulobacter
Blautia
Actinomyces
Porphyromonas
Gemmiger
Neisseria
Rothia
Sediminibacterium
Ochrobactrum
Bifidobacterium
Lactobacillus
Sphingomonas
Veillonella
Prevotella
Acinetobacter
Vogesella
Cupriavidus
Streptococcus
B
Relative Abundance (%)
0
20
40
60
b
m
s
u
f
Top 20 genus
Agrobacterium
Bifidobacterium
Fusobacterium
Dialister
Veillonella
Porphyromonas
Bacteroides
Faecalibacterium
Actinomyces
Neisseria
Roseburia
Acinetobacter
Haemophilus
Gemmiger
Lactobacillus
Prevotella
Rothia
Sediminibacterium
Ochrobactrum
Streptococcus
C
Relative Abundance (%)
0
25
50
75
B
M
S
U
F
Top 20 phylum
Gemmatimonadetes
Synergistetes
GN02
Nitrospirae
Planctomycetes
Chloroflexi
Cyanobacteria
[Thermi]
OD1
Tenericutes
SR1
Acidobacteria
Spirochaetes
Verrucomicrobia
TM7
Fusobacteria
Actinobacteria
Bacteroidetes
Firmicutes
Proteobacteria
D
Relative Abundance (%)
0
25
50
75
100
b
m
s
u
f
Top 20 phylum
Armatimonadetes
Gemmatimonadetes
Chlamydiae
WPS-2
Verrucomicrobia
Chloroflexi
SR1
Tenericutes
Spirochaetes
Planctomycetes
Acidobacteria
OD1
Cyanobacteria
TM7
[Thermi]
Fusobacteria
Actinobacteria
Bacteroidetes
Proteobacteria
Firmicutes

Figure S2. The microbial compositions of samples from ESCC patients and ESCC patients. (A) In blood samples, the Simpson, Pielou, and Faith indexes were higher in healthy subjects than in ESCC patients. (B) In oral mucosal samples, the Faith index was higher in healthy subjects than in ESCC patients. (C) In saliva samples, the Chao1, Faith, and Good’s coverage indexes were higher in healthy subjects than in ESCC patients. (D) In urine samples, the Chao1, Pielou, observed species, and Faith indexes were higher in healthy subjects than in ESCC patients. (E) In fecal samples, the Chao1 index was higher in healthy subjects than in ESCC patients, whereas the Good’s coverage index was lower in healthy subjects than in ESCC patients. The number of biological replicates performed was as follows: in ESCC patients, B (n =20), M (n=24), S (n=23), U (n=21), F (n=22); in healthy subjects, b (n =27), m (n=26), s (n=26), u (n=25), f (n=22).

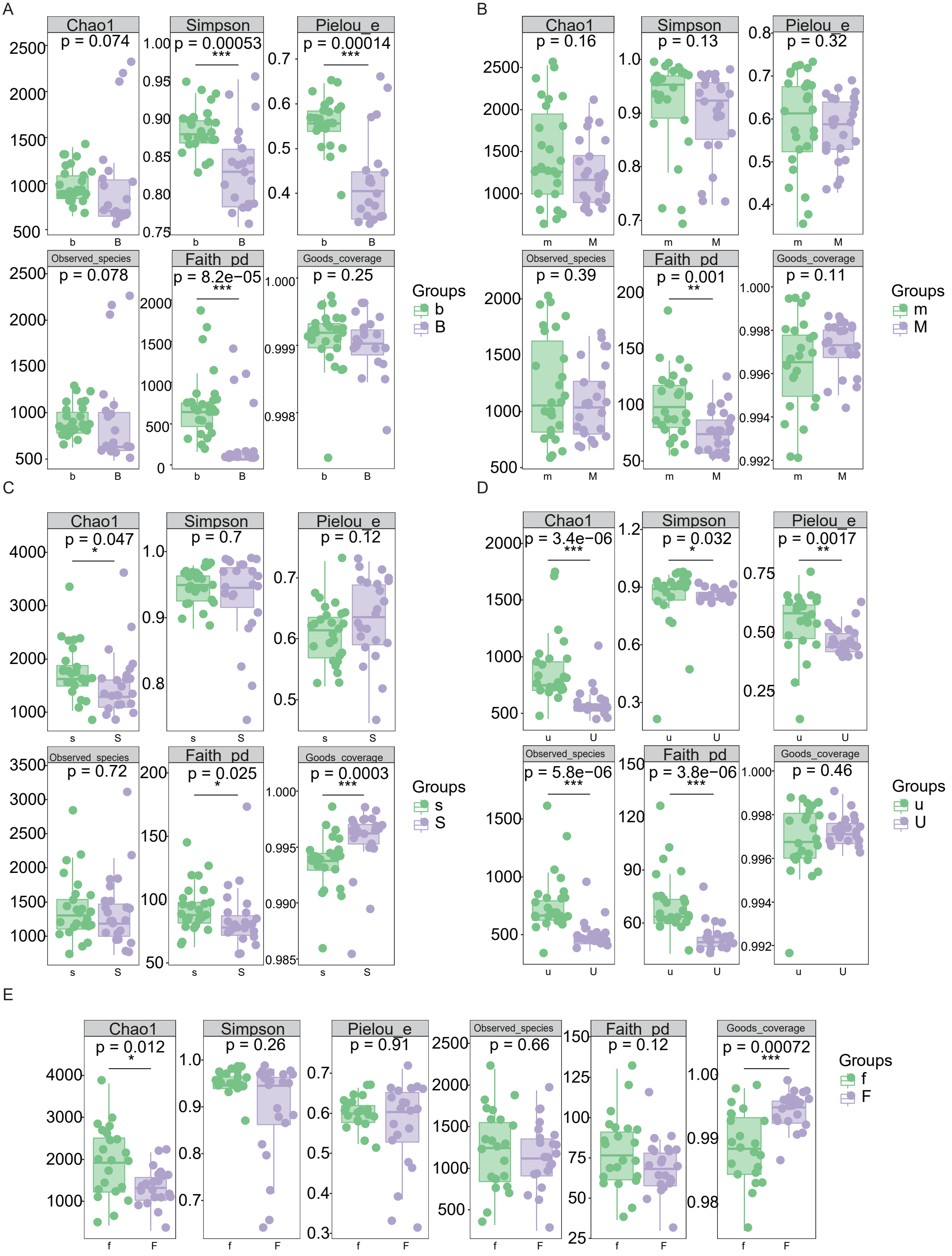
A
Chao1
p = 0.074
Simpson
p = 0.00053
***
Pielou_e
p = 0.00014
***
Observed_species
p = 0.078
Faith_pd
p = 8.2e−05
***
Goods_coverage
p = 0.25
Groups
b
B
B
Chao1
p = 0.16
Simpson
p = 0.13
Pielou_e
p = 0.32
Observed_species
p = 0.39
Faith_pd
p = 0.001
**
Goods_coverage
p = 0.11
Groups
m
M
C
Chao1
p = 0.047
*
Simpson
p = 0.7
Pielou_e
p = 0.12
Observed_species
p = 0.72
Faith_pd
p = 0.025
*
Goods_coverage
p = 0.0003
***
Groups
s
S
D
Chao1
p = 3.4e−06
***
Simpson
p = 0.032
*
Pielou_e
p = 0.0017
**
Observed_species
p = 5.8e−06
***
Faith_pd
p = 3.8e−06
***
Goods_coverage
p = 0.46
Groups
u
U
E
Chao1
p = 0.012
*
Simpson
p = 0.26
Pielou_e
p = 0.91
Observed_species
p = 0.66
Faith_pd
p = 0.12
Goods_coverage
p = 0.00072
***
Groups
f
F

Figure S3. The predicted KEGG secondary functional pathways of samples from ESCC patients and healthy subjects are different. (A) Abundance map of predicted KEGG secondary functional pathways in ESCC patients suggests that metabolic pathways accounted for the majority of pathways. (B) Abundance map of predicted KEGG secondary functional pathways in healthy subjects suggests that metabolic pathways accounted for the majority of pathways. (C) In blood samples, the metabolic pathways of microbes were significantly different between ESCC patients and healthy subjects. (D) In oral mucosal samples, only the betalain-biosynthesis metabolic pathway was upregulated in ESCC patients compared to healthy subjects. (E) In saliva samples, the betalain-biosynthesis and limonene-and-pinene-degradation metabolic pathways were upregulated in ESCC patients compared to healthy subjects. (F) In urine samples, the indole-alkaloid biosynthesis metabolic pathways was upregulated in ESCC patients compared to healthy subjects. The number of biological replicates performed was as follows: in ESCC patients, B (*n* =20), M (*n*=24), S (*n*=23), U (*n*=21), F (*n*=22); in healthy subjects, b (*n* =27), m (*n*=26), s (*n*=26), u (*n*=25), f (*n*=22).

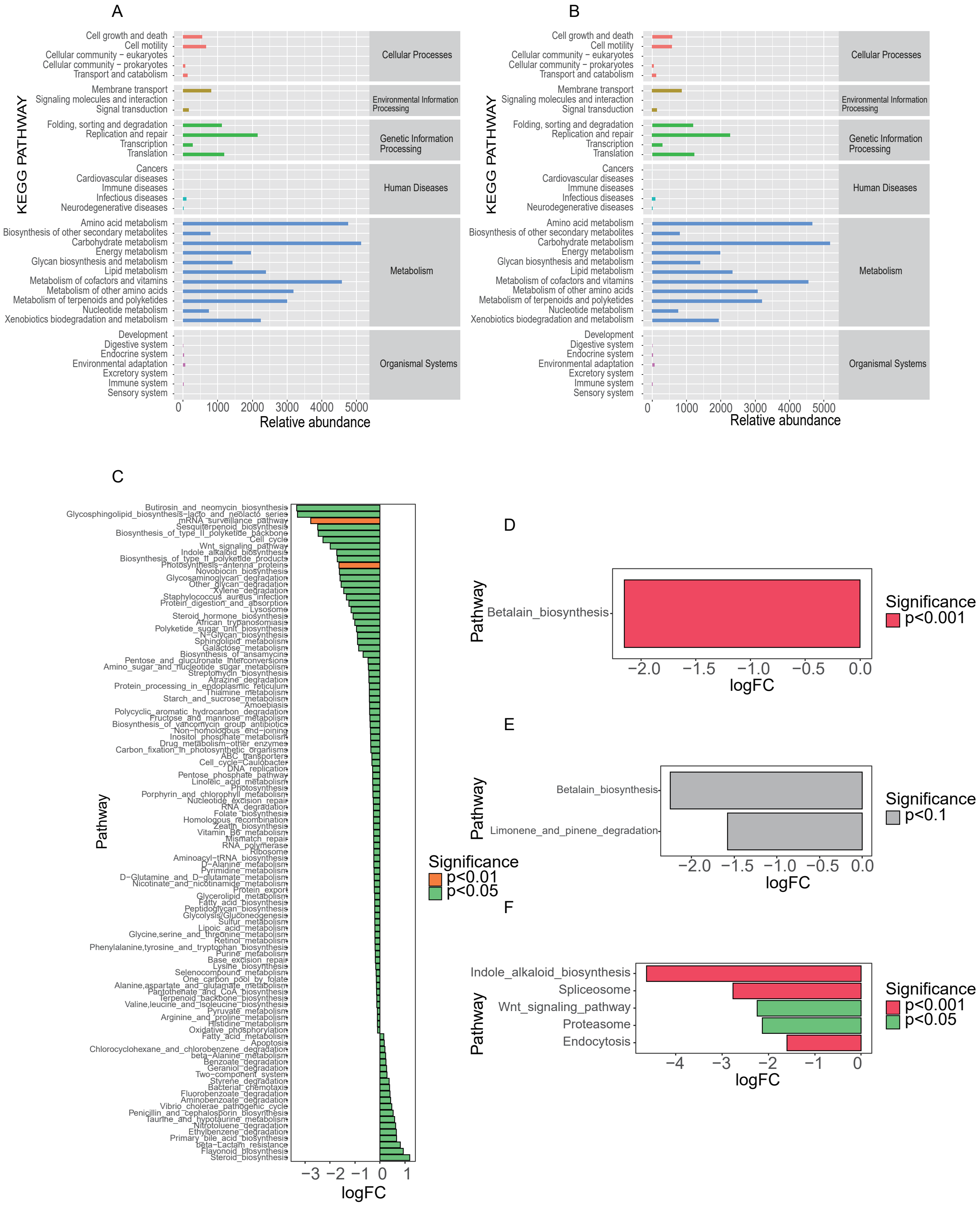
A
B
KEGG PATHWAY
Cell growth and death
Cell motility
Cellular community − eukaryotes
Cellular community − prokaryotes
Transport and catabolism
Membrane transport
Signaling molecules and interaction
Signal transduction
Folding, sorting and degradation
Replication and repair
Transcription
Translation
Cancers
Cardiovascular diseases
Immune diseases
Infectious diseases
Neurodegenerative diseases
Amino acid metabolism
Biosynthesis of other secondary metabolites
Carbohydrate metabolism
Energy metabolism
Glycan biosynthesis and metabolism
Lipid metabolism
Metabolism of cofactors and vitamins
Metabolism of other amino acids
Metabolism of terpenoids and polyketides
Nucleotide metabolism
Xenobiotics biodegradation and metabolism
Development
Digestive system
Endocrine system
Environmental adaptation
Excretory system
Immune system
Sensory system
Cellular Processes
Environmental Information Processing
Genetic Information Processing
Human Diseases
Metabolism
Organismal Systems
0 1000 2000 3000 4000 5000
Relative abundance
C
Pathway
Butirosin_and_neomycin_biosynthesis
Glycosphingolipid_biosynthesis−lacto_and_neolacto_series
mRNA_surveillance_pathway
Sesquiterpenoid_biosynthesis
Biosynthesis_of_type_II_polyketide_backbone
Cell_cycle
Wnt_signaling_pathway
Indole_alkaloid_biosynthesis
Biosynthesis_of_type_II_polyketide_products
Photosynthesis−antenna_proteins
Novobiocin_biosynthesis
Glycosaminoglycan_degradation
Other_glycan_degradation
Xylene_degradation
Staphylococcus_aureus_infection
Protein_digestion_and_absorption
Lysosome
Steroid_hormone_biosynthesis
African_trypanosomiasis
Polyketide_sugar_unit_biosynthesis
N−Glycan_biosynthesis
Sphingolipid_metabolism
Galactose_metabolism
Biosynthesis_of_ansamycins
Pentose_and_glucuronate_interconversions
Amino_sugar_and_nucleotide_sugar_metabolism
Streptomycin_biosynthesis
Atrazine_degradation
Protein_processing_in_endoplasmic_reticulum
Thiamine_metabolism
Starch_and_sucrose_metabolism
Amoebiasis
Polycyclic_aromatic_hydrocarbon_degradation
Fructose_and_mannose_metabolism
Biosynthesis_of_vancomycin_group_antibiotics
Non−homologous_end−joining
Inositol_phosphate_metabolism
Drug_metabolism−other_enzymes
Carbon_fixation_in_photosynthetic_organisms
ABC_transporters
Cell_cycle−Caulobacter
DNA_replication
Pentose_phosphate_pathway
Linoleic_acid_metabolism
Photosynthesis
Porphyrin_and_chlorophyll_metabolism
Nucleotide_excision_repair
RNA_degradation
Folate_biosynthesis
Homologous_recombination
Zeatin_biosynthesis
Vitamin_B6_metabolism
Mismatch_repair
RNA_polymerase
Ribosome
Aminoacyl−tRNA_biosynthesis
D−Alanine_metabolism
Pyrimidine_metabolism
D−Glutamine_and_D−glutamate_metabolism
Nicotinate_and_nicotinamide_metabolism
Protein_export
Glycerolipid_metabolism
Fatty_acid_biosynthesis
Peptidoglycan_biosynthesis
Glycolysis/Gluconeogenesis
Sulfur_metabolism
Lipoic_acid_metabolism
Glycine,serine_and_threonine_metabolism
Retinol_metabolism
Phenylalanine,tyrosine_and_tryptophan_biosynthesis
Purine_metabolism
Base_excision_repair
Lysine_biosynthesis
Selenocompound_metabolism
One_carbon_pool_by_folate
Alanine,aspartate_and_glutamate_metabolism
Pantothenate_and_CoA_biosynthesis
Terpenoid_backbone_biosynthesis
Valine,leucine_and_isoleucine_biosynthesis
Pyruvate_metabolism
Arginine_and_proline_metabolism
Histidine_metabolism
Oxidative_phosphorylation
Fatty_acid_metabolism
Apoptosis
Chlorocyclohexane_and_chlorobenzene_degradation
beta−Alanine_metabolism
Benzoate_degradation
Geraniol_degradation
Two−component_system
Styrene_degradation
Bacterial_chemotaxis
Fluorobenzoate_degradation
Aminobenzoate_degradation
Vibrio_cholerae_pathogenic_cycle
Penicillin_and_cephalosporin_biosynthesis
Taurine_and_hypotaurine_metabolism
Nitrotoluene_degradation
Ethylbenzene_degradation
Primary_bile_acid_biosynthesis
beta−Lactam_resistance
Flavonoid_biosynthesis
Steroid_biosynthesis
−3 −2 −1 0 1
logFC
Significance
p<0.01
p<0.05
D
Pathway
Betalain_biosynthesis
−2.0 −1.5 −1.0 −0.5 0.0
logFC
Significance
p<0.001
E
Pathway
Betalain_biosynthesis
Limonene_and_pinene_degradation
−2.0 −1.5 −1.0 −0.5 0.0
logFC
Significance
p<0.1
F
Pathway
Indole_alkaloid_biosynthesis
Spliceosome
Wnt_signaling_pathway
Proteasome
Endocytosis
−4 −3 −2 −1 0
logFC
Significance
p<0.001
p<0.05

Figure S4. The microbial compositions of samples from ESCC patients after chemotherapy and ESCC patients after immunotherapy. (A) The microbial composition of patients with ESCC after chemotherapy in blood, oral mucosal, saliva, urine, and fecal samples at the genus level. (B) The microbial composition of patients with ESCC after immunotherapy in blood, oral mucosal, saliva, urine, and fecal samples at the genus level. (C) The composition of the microbiota in the blood (BI), oral mucosa (MI), saliva (SI), urine (UI) and feces (FI) of patients with ESCC after immunotherapy at the phylum level; the top twenty kinds of microbes at the phylum level in patients with ESCC after immunotherapy. (D) The composition of the microbiota in the blood (BH), oral mucosa (MH), saliva (SH), urine (UH), and feces (FH) of patients with ESCC after chemotherapy at the phylum level; the top twenty kinds of microbes at the phylum level in patients with ESCC after chemotherapy. The number of biological replicates is as follows: in patients with ESCC after chemotherapy, BH(n =8), MH(n=14), SH(n=14), UH(n=13), FH(n=14); in patients with ESCC after immunotherapy, BI(n=27), MI(n=27), SI(n=27), UI(n=27), FI(n=22).

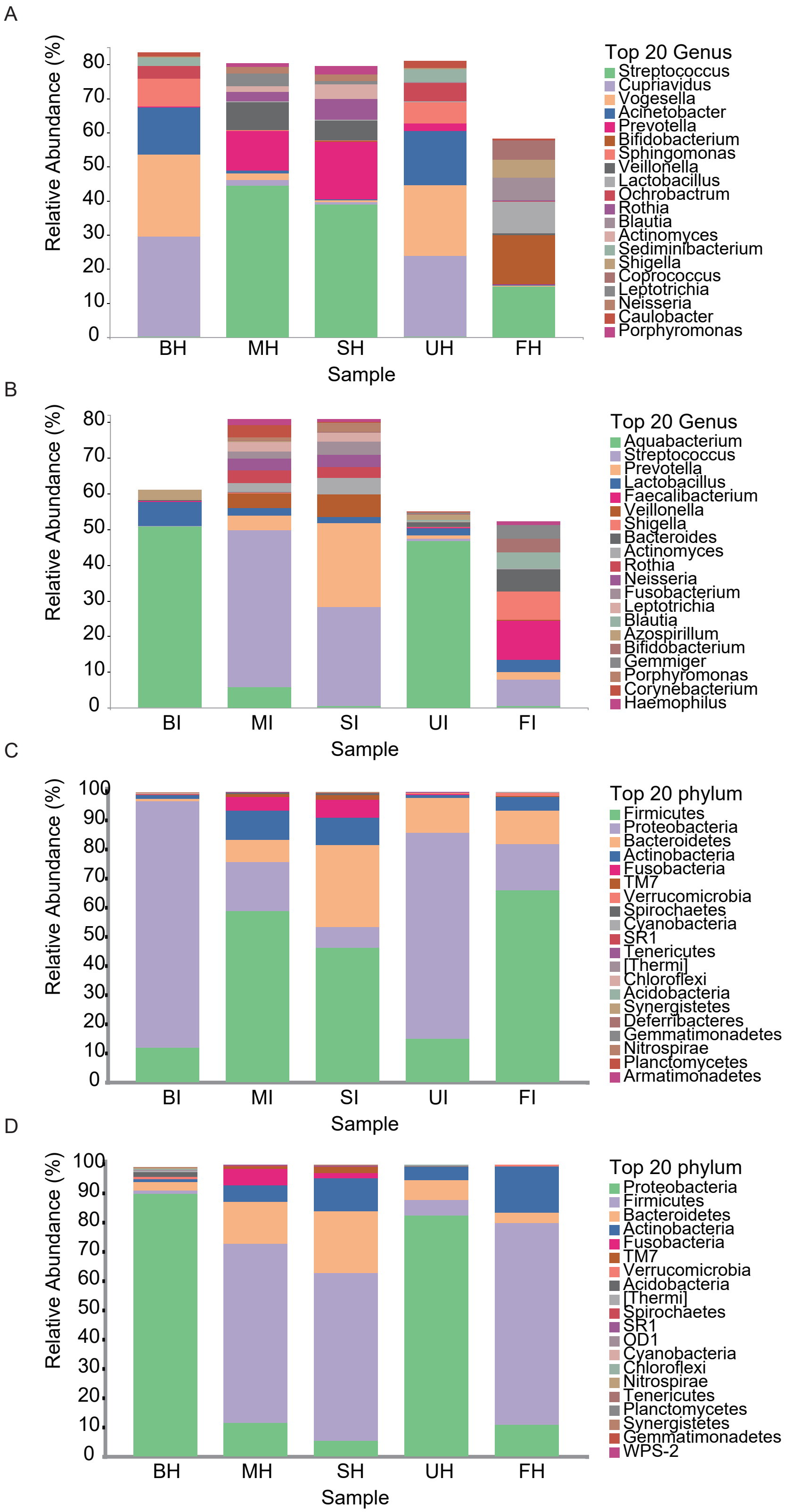

A
Relative Abundance (%)
0
10
20
30
40
50
60
70
80
BH
MH
SH
UH
FH
Sample
Top 20 Genus
Streptococcus
Cupriavidus
Vogesella
Acinetobacter
Prevotella
Bifidobacterium
Sphingomonas
Veillonella
Lactobacillus
Ochrobactrum
Rothia
Blautia
Actinomyces
Sediminibacterium
Shigella
Coprococcus
Leptotrichia
Neisseria
Caulobacter
Porphyromonas
B
Relative Abundance (%)
BI
MI
SI
UI
FI
Sample
Top 20 Genus
Aquabacterium
Streptococcus
Prevotella
Lactobacillus
Faecalibacterium
Veillonella
Shigella
Bacteroides
Actinomyces
Rothia
Neisseria
Fusobacterium
Leptotrichia
Blautia
Azospirillum
Bifidobacterium
Gemmiger
Porphyromonas
Corynebacterium
Haemophilus
C
Relative Abundance (%)
100
90
BI
MI
SI
UI
FI
Sample
Top 20 phylum
Firmicutes
Proteobacteria
Bacteroidetes
Actinobacteria
Fusobacteria
TM7
Verrucomicrobia
Spirochaetes
Cyanobacteria
SR1
Tenericutes
[Thermi]
Chloroflexi
Acidobacteria
Synergistetes
Deferribacteres
Gemmatimonadetes
Nitrospirae
Planctomycetes
Armatimonadetes
D
Relative Abundance (%)
BH
MH
SH
UH
FH
Sample
Top 20 phylum
Proteobacteria
Firmicutes
Bacteroidetes
Actinobacteria
Fusobacteria
TM7
Verrucomicrobia
Acidobacteria
[Thermi]
Spirochaetes
SR1
OD1
Cyanobacteria
Chloroflexi
Nitrospirae
Tenericutes
Planctomycetes
Synergistetes
Gemmatimonadetes
WPS-2

Figure S5. The microbial compositions of samples from ESCC patients after chemotherapy and ESCC patients after immunotherapy are highly different. (A) In blood samples, the Faith index of patients with ESCC after immunotherapy was higher than those in samples from patients with ESCC after chemotherapy. (B) In oral mucosal samples, the Good's coverage index of patients with ESCC after immunotherapy was higher than that of patients with ESCC after chemotherapy. The Chao1 and observed species indexes of patients with ESCC after immunotherapy was less than that of patients with ESCC after chemotherapy. (C) In saliva samples, there is no statistically significant difference in α diversity between patients with ESCC after immunotherapy and patients with ESCC after chemotherapy. (D) In urine samples, the Chao1, Pielou, observed species, and Faith indexes of patients with ESCC after immunotherapy were higher than those of patients with ESCC after chemotherapy. (E) In fecal samples, there is no statistically significant difference in αdiversity between patients with ESCC after immunotherapy and patients with ESCC after chemotherapy. The number of biological replicates is as follows: in patients with ESCC after chemotherapy, BH(n =8), MH(n=14), SH(n=14), UH(n=13), FH(n=14); in patients with ESCC after immunotherapy, BI(n=27), MI(n=27), SI(n=27), UI(n=27), FI(n=22).

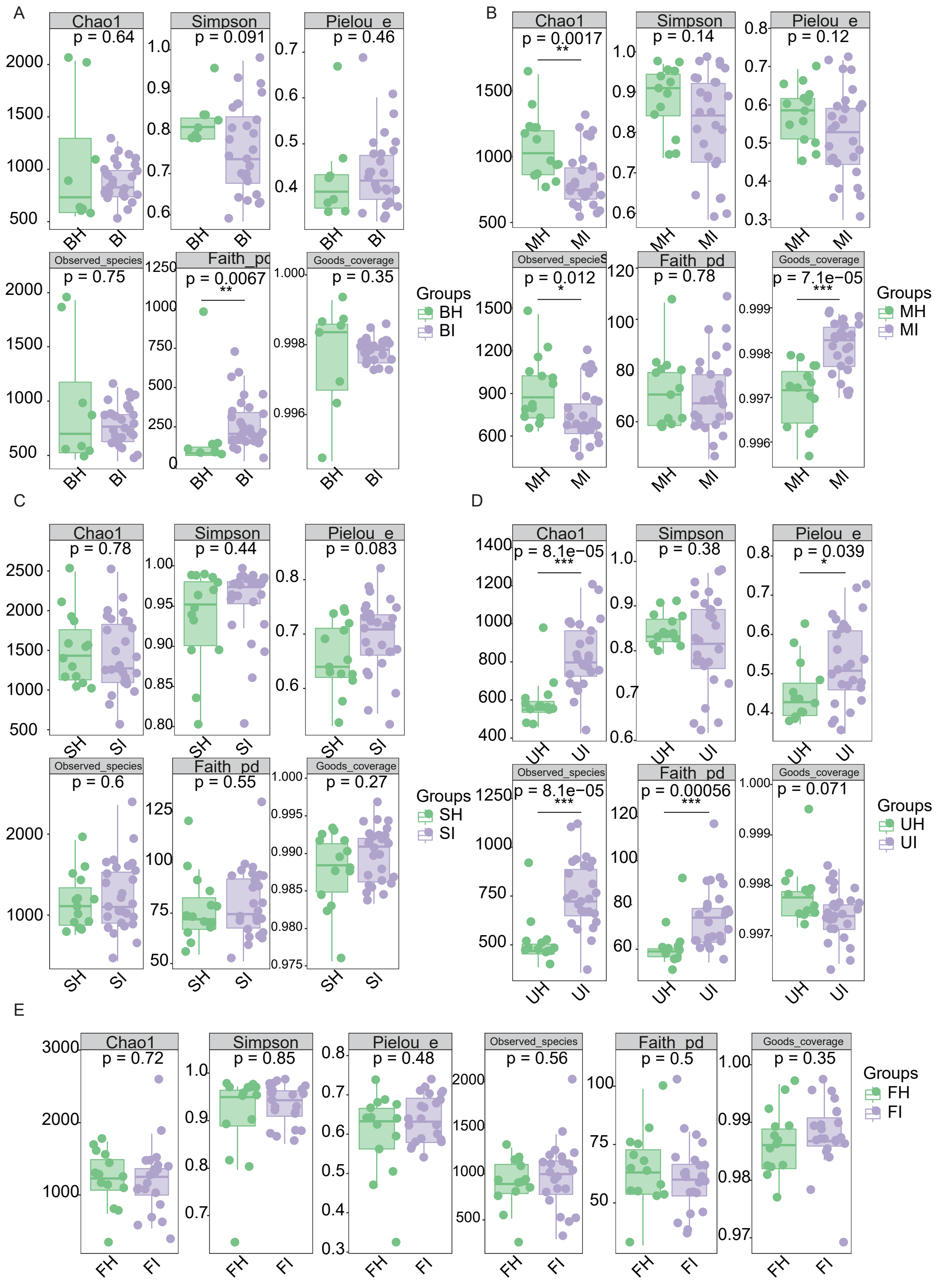
A
Chao1
p = 0.64
Simpson
p = 0.091
Pielou_e
p = 0.46
BH
BI
Observed_species
p = 0.75
Faith_pd
p = 0.0067
**
Goods_coverage
p = 0.35
Groups
BH
BI
B
Chao1
p = 0.0017
**
Simpson
p = 0.14
Pielou_e
p = 0.12
MH
MI
Observed_species
p = 0.012
*
Faith_pd
p = 0.78
Goods_coverage
p = 7.1e−05
***
Groups
MH
MI
C
Chao1
p = 0.78
Simpson
p = 0.44
Pielou_e
p = 0.083
SH
SI
Observed_species
p = 0.6
Faith_pd
p = 0.55
Goods_coverage
p = 0.27
Groups
SH
SI
D
Chao1
p = 8.1e−05
***
Simpson
p = 0.38
Pielou_e
p = 0.039
*
UH
UI
Observed_species
p = 8.1e−05
***
Faith_pd
p = 0.00056
***
Goods_coverage
p = 0.071
Groups
UH
UI
E
Chao1
p = 0.72
Simpson
p = 0.85
Pielou_e
p = 0.48
Observed_species
p = 0.56
Faith_pd
p = 0.5
Goods_coverage
p = 0.35
FH
FI
Groups
FH
FI

Figure S6. The predicted KEGG secondary functional pathways of samples from ESCC patients after chemotherapy and ESCC patients after immunotherapy are different. (A) Abundance map of predicted KEGG secondary functional pathways in ESCC patients after chemotherapy suggests that metabolic pathways accounted for the majority of pathways. (B) Abundance map of predicted KEGG secondary functional pathways in ESCC patients after immunotherapy suggests that metabolic pathways accounted for the majority of pathways. (C) In blood samples, the metabolic pathways of microbes were significantly different between ESCC patients after chemotherapy and ESCC patients after immunotherapy. (D) In oral mucosal samples, the polyketide sugar unit biosynthesis and betalain biosynthesis metabolic pathway was underrepresented in patients with ESCC after immunotherapy compared with that in patients with ESCC after chemotherapy. (E) In saliva samples, the photosynthesis and lysosome metabolic pathways were overrepresented in patients with ESCC after immunotherapy. (F) In urine samples, the metabolic pathways of microbes were significantly different between ESCC patients after chemotherapy and ESCC patients after immunotherapy. (G) In fecal samples, the tetracycline biosynthesis, Lysosome, and shigellosis metabolic pathway was overrepresented in patients with ESCC after immunotherapy compared with that in patients with ESCC after chemotherapy. The number of biological replicates is as follows: in patients with ESCC after chemotherapy, BH(n =8), MH(n=14), SH(n=14), UH(n=13), FH(n=14); in patients with ESCC after immunotherapy, BI(n=27), MI(n=27), SI(n=27), UI(n=27), FI(n=22).

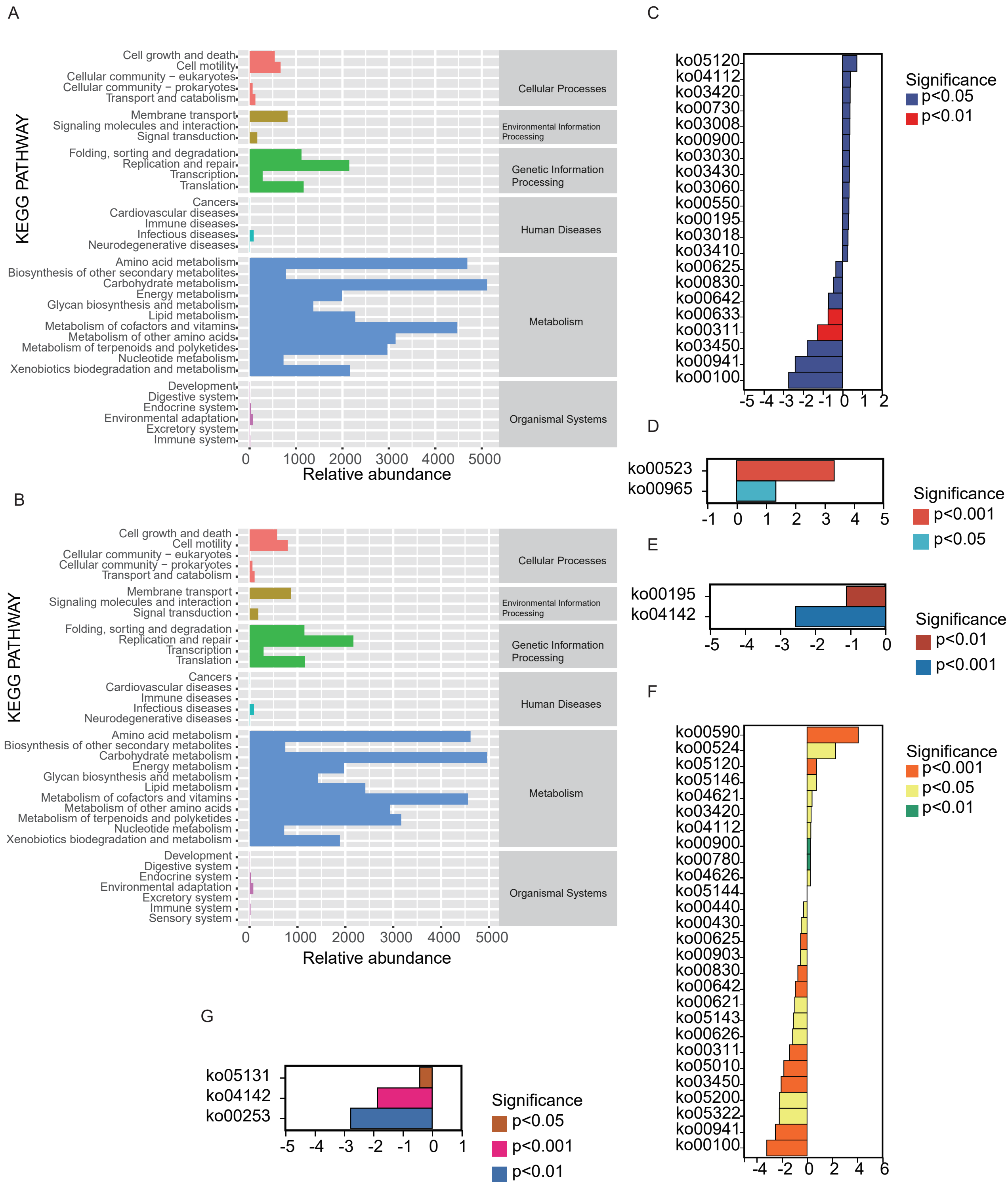
A
B
C
D
E
F
G
KEGG PATHWAY
Cell growth and death
Cell motility
Cellular community − eukaryotes
Cellular community − prokaryotes
Transport and catabolism
Membrane transport
Signaling molecules and interaction
Signal transduction
Folding, sorting and degradation
Replication and repair
Transcription
Translation
Cancers
Cardiovascular diseases
Immune diseases
Infectious diseases
Neurodegenerative diseases
Amino acid metabolism
Biosynthesis of other secondary metabolites
Carbohydrate metabolism
Energy metabolism
Glycan biosynthesis and metabolism
Lipid metabolism
Metabolism of cofactors and vitamins
Metabolism of other amino acids
Metabolism of terpenoids and polyketides
Nucleotide metabolism
Xenobiotics biodegradation and metabolism
Development
Digestive system
Endocrine system
Environmental adaptation
Excretory system
Immune system
Sensory system
Cellular Processes
Environmental Information Processing
Genetic Information Processing
Human Diseases
Metabolism
Organismal Systems
0 1000 2000 3000 4000 5000
Relative abundance
ko05120 ko04112 ko03420 ko00730 ko03008 ko00900 ko03030 ko03430 ko03060 ko00550 ko00195 ko03018 ko03410 ko00625 ko00830 ko00642 ko00633 ko00311 ko03450 ko00941 ko00100
-5 -4 -3 -2 -1 0 1 2
Significance
p<0.05
p<0.01
ko00523
ko00965
-1 0 1 2 3 4 5
Significance
p<0.001
p<0.05
ko00195
ko04142
-5 -4 -3 -2 -1 0
Significance
p<0.01
p<0.001
ko00590 ko00524 ko05120 ko05146 ko04621 ko03420 ko04112 ko00900 ko00780 ko04626 ko05144 ko00440 ko00430 ko00625 ko00903 ko00830 ko00642 ko00621 ko05143 ko00626 ko00311 ko05010 ko03450 ko05200 ko05322 ko00941 ko00100
-4 -2 0 2 4 6
Significance
p<0.001
p<0.05
p<0.01
ko05131
ko04142
ko00253
-5 -4 -3 -2 -1 0 1
Significance
p<0.05
p<0.001
p<0.01
